# MicroRNA-574-5p Was Pivotal for TLR9 Signaling Enhanced Tumor Progression via Down-Regulating Checkpoint Suppressor 1 in Human Lung Cancer

**DOI:** 10.1371/journal.pone.0048278

**Published:** 2012-11-02

**Authors:** Qinchuan Li, Xiaoman Li, Zhongliang Guo, Feng Xu, Jingyan Xia, Zhongmin Liu, Tao Ren

**Affiliations:** 1 Department of Cardiothoracic Surgery, East Hospital, Tongji University School of Medicine, Shanghai, China; 2 Department of Clinical Laboratory, Shanghai Ninth People’s Hospital, Shanghai Jiao Tong University School of Medicine, Shanghai, China; 3 Department of Respiratory Medicine, East Hospital, Tongji University School of Medicine, Shanghai, China; 4 Department of Respiratory Medicine, Second Affiliated Hospital, Zhejiang University School of Medicine, Hangzhou, China; 5 Department of Radiation Therapy, Second Affiliated Hospital, Zhejiang University School of Medicine, Hangzhou, China; The University of Texas M.D Anderson Cancer Center, United States of America

## Abstract

Accumulating data suggested that functional expression of Toll-like receptors (TLRs) in tumor cells was involved in tumor progression. Our previous study demonstrated that TLR9 signaling could enhance the tumor progression of human lung cancer cells in vitro and in vivo. We further showed that miR-574-5p was the mostly up-regulated miRNA in human lung cancer cells under TLR9 signaling by miRNA array analysis. Here we characterized the potential role of miRNA-574-5p in enhanced tumor progression induced by TLR9 signaling in human lung cancer. We confirmed that TLR9 signaling effectively elevated the expression of miR-574-5p in human lung cancer cells. Notably, we found that down-regulation of miRNA-574-5p using miR-574-5p inhibitor in vitro or miR-574-5p sponge in vivo significantly abrogated the enhanced tumor progression induced by TLR9 signaling. Further studies showed that miR-574-5p was an important player associated with enhanced tumor progression of human lung cancer cells. Notably, we identified checkpoint suppressor 1 (Ches1) as the dominant direct target for miRNA-574-5p to confer the TLR9 signaling enhanced tumor progression. We revealed that over-expression of Ches1 significantly inhibited the cell cycle entry of human lung cancer cells. Finally, we revealed that the expression of miR-574-5p was positively correlated with TLR9 and reversely correlated with Ches1 in lung cancer patients. Our findings not only facilitated the further understanding of the crosstalk between miRNAs and TLRs in tumor biology, but also provided novel potential candidates for treatment of cancer.

## Introduction

The discovery of a series of innate immune-specific receptors activated by pathogen-associated molecular patterns led to a new understanding of innate immunity mechanisms. Among the innate immune-specific receptors, the best characterized are the Toll-like receptors (TLRs), which recognize a variety of pathogen-associated molecular patterns, are mainly expressed on the immune cells and play an important role in innate and adaptive immunity [1]–[3]. Interestingly, a growing body of literature demonstrated that functional TLRs were also widely expressed on a variety of tumor cells including breast, brain, gastric and lung cancer cells [4]. Accumulating data showed that TLRs agonist could promote the invasion and enhance the metastatic potential of tumor cells, indicating that activation of TLRs signaling in tumor cells was involved in tumor progress [5]–[8]. Our previous study demonstrated that CpG oligodeoxynucleotides (CpG ODNs), which were under investigation as adjuvant in therapy against infections and cancers, could effectively activate the TLR9 signaling pathway in human lung cancer cells and thus promoted the tumor progression both in vitro and in vivo [3], [4], [9]–[11]. We further showed that up-regulation of cyclin-dependent kinase 2 (CDK2) was critical for TLR9 signaling to stimulate the proliferation and cell cycle entry of human lung cancer cells [4]. However, the precise mechanisms for how TLR9 signaling was involved in tumor progression of human lung cancer were still far less clear.

MicroRNAs (miRNAs) have emerged as a major class of gene expression regulators, post-transcriptionally regulating gene expression through base pairing to partially complementary sites to prevent protein accumulation by repressing translation or by inducing mRNA degradation, linked to most biological functions including tumor biology [12], [13]. Accumulating data suggested that miRNAs were effective and novel biomarkers for lung cancer diagnosis, prediction and treatment [14], [15]. Meanwhile, miRNAs were also implicated in regulating the biological effects of TLRs signaling [16]–[18]. To investigate the potential role of miRNAs in the increased tumor progression of human lung cancer cells upon TLR9 agonist stimulation, we performed miRNA microarray assay to detect the expression profile of miRNA in 95D cells with or without treatment of CpG ODNs, and found that CpG ODNs stimulation alternated the miRNA expression profile in 95D cells and the difference in expressions of 23 miRNAs between the CpG ODNs treated group and untreated group was at least two-fold, among which miRNA-574-5p was the mostly up-regulated miRNA in CpG ODNs stimulated 95D cells compared with that the untreated group [19]. However, the possible effect of miRNA-574-5p on the enhanced tumor progression induced by TLR9 signaling still remains to be elucidated.

To address this issue, here we characterized the effect of miRNA-574-5p which was up-regulated under TLR9 signaling in human lung cancer cells. We found that miRNA-574-5p was pivotal for TLR9 signaling to enhance the tumor progression of human lung cancer cells. Further studies showed that checkpoint suppressor 1 (Ches1) was a dominant direct target for miRNA-574-5p to confer the TLR9 signaling enhanced tumor progression. These findings extended previous studies and provided a novel insight into the understanding of miRNAs in the functional expression of TLR9 in tumor cells.

**Table 1:** The clinical pathological characters of the NSCLC patients.

Clinical pathological parameter	Number
Sex	
Male	16
Female	7
Age (years)	51–73
Tumor stage	
T1/2	3
T3/4	20
Nodal status	
N0/1	6
N2/3	17
Histological type	
Adenocarcinoma	15
Others	8

1 Lymph nodal metastasis is according to pathological diagnosis and clinical palpation.

2 Clinical stage is according to TNM stage.

**Figure 1:**
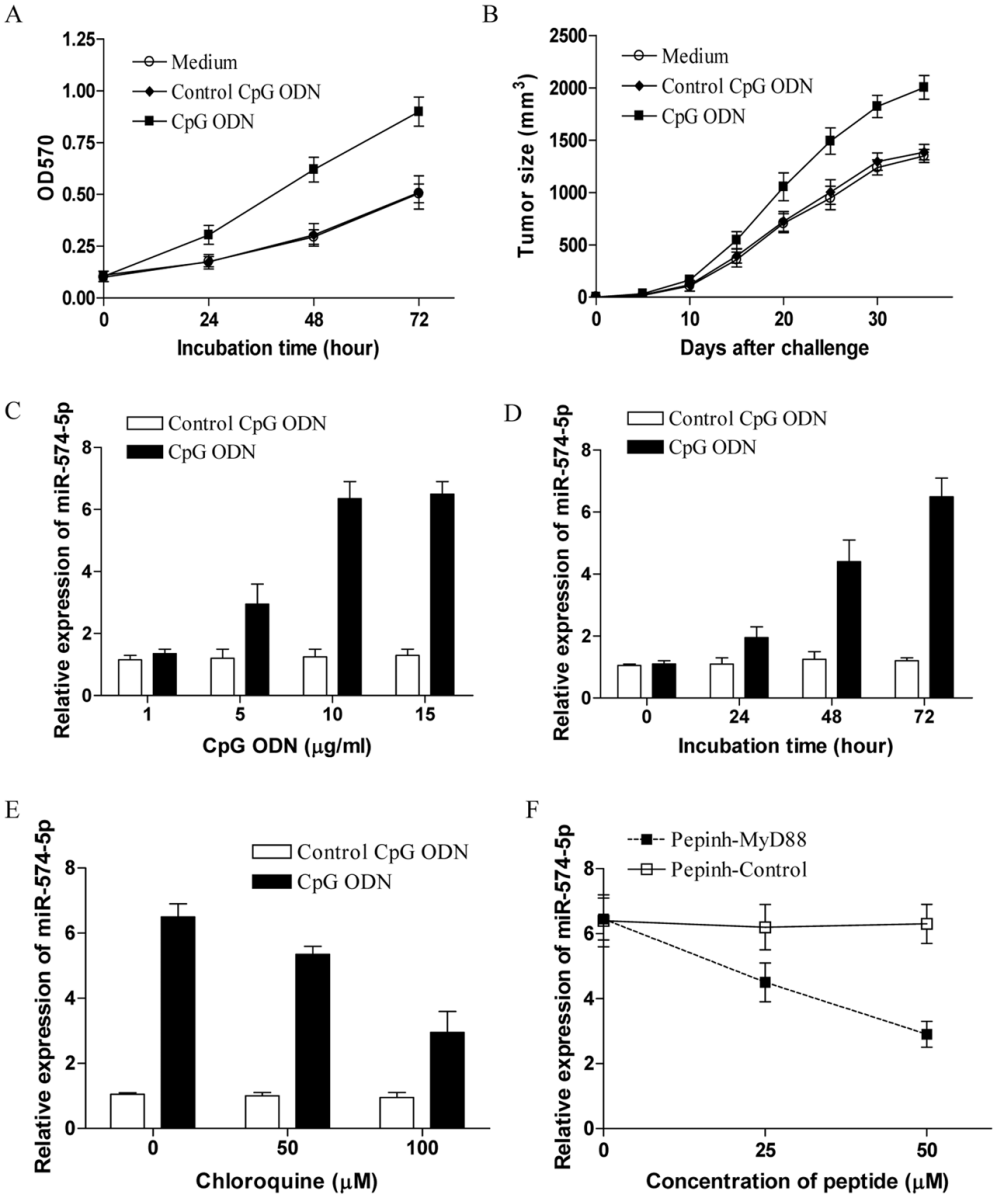
Up-regulation of miR-574-5p in TLR9 signaling stimulated human lung cancer cells. (A) 95D cells were treated with 10 µg/ml CpG ODNs or control CpG ODNs for the indicated time and detected for their proliferation. (B) Groups of eight nude mice were challenged with 2×10^6^ of 95D cells. Five days later, the tumor bearing mice were injected in situ with 100 µg of CpG ODNs at 7 day intervals. The control group received equal dose of the control CpG ODNs or the equal volume of the medium. The tumor size was determined. Each bar represents the means (±SD) from eight nude mice in each group. (C) 95D cells were treated with the indicated dose of CpG ODNs for 72 h and then assayed for their expression of miR-574-5p. (D) 95D cells were treated with 10 µg/ml CpG ODNs for the indicated time and assayed for their expression of miR-574-5p. (E) 95D cells were treated with 10 µg/ml CpG ODNs in the presence of the indicated dose of chloroquine for 72 h and then assayed for their expression of miR-574-5p. (F) 95D cells were treated with 10 µg/ml CpG ODNs in the presence of the indicated dose of MyD88 inhibitory peptide (Pepinh-MyD88) or the control peptide (Pepinh-Control) for 72 h and then assayed for their expression of miR-574-5p. Error bars indicated standard deviation of triplicate measurements from three independent experiments.

## Materials and Methods

### Ethics Statement

All patients in this study were given written informed consent, and the human study was approved by the Ethics Committee of Tongji University (Permit number: 20090128). All animal experiments were performed according to the Guide for the Care and Use of Medical Laboratory Animals and with the ethical approval of Shanghai Medical Laboratory Animal Care and Use Committee as well as the Ethical Committee of Tongji University (Permit number: 20110016).

**Figure 2:**
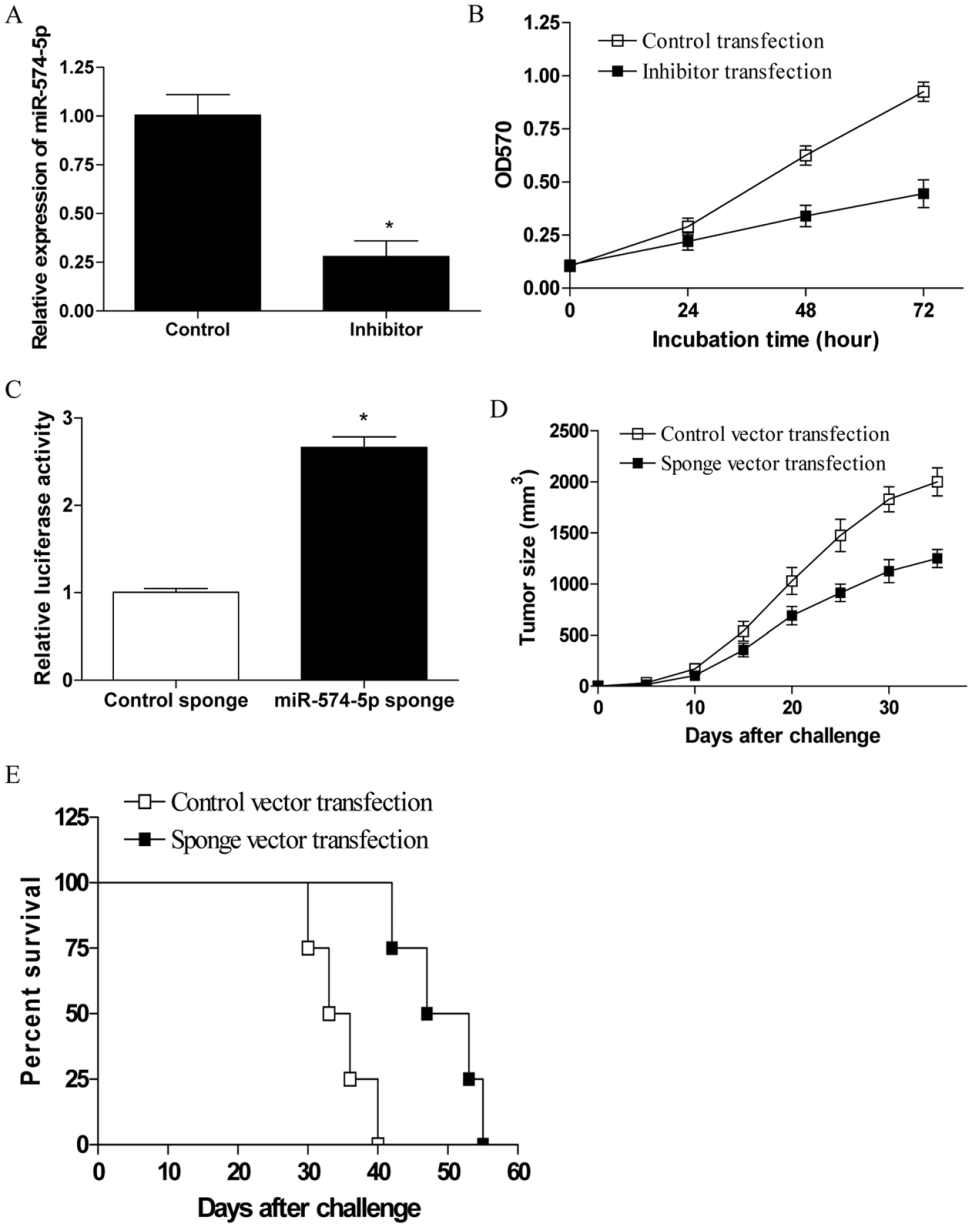
Down-regulation of miR-574-5p abrogated the TLR9 signaling enhanced tumor progression. (A) 95D cells were transfected with miR-574-5p inhibitor for 48 h and then assayed for their expression of miR-574-5p. (B) 95D cells were transfected with miR-574-5p inhibitor or the control respectively, and then stimulated with 10 µg/ml CpG ODNs for the indicated time. Error bars indicated standard deviation of triplicate measurements form three independent experiments. (C) Enhanced activity of miR-574-5p-regulated reporter was achieved by infection of 95D cells with the miR-574-5p sponge. (D and E) Groups of eight nude mice were challenged with 2×10^6^ of 95D cells which were stably transfected with miR-574-5p sponge vector or the control vector. Five days later, the tumor bearing mice were injected in situ with 100 µg of CpG ODNs at 7 day intervals. The tumor size and the survival time of tumor bearing mice were determined. Each bar represents the means (±SD) from eight nude mice in each group. *p<0.05.

### Patients

Between June 2009 and March 2012, we collected tumor samples from patients with lung cancer in East Hospital, Shanghai, China. The study group (n = 23) comprised chemotherapy and radiotherapy naive patients with lung cancer. Review of pathology reports confirmed the diagnosis. Subjects with autoimmune diseases (e.g. rheumatoid arthritis, systemic lupus erythematosus), chronic infections (e.g. human immunodeficiency virus infection, tuberculosis), bone marrow involvement, anticoagulant and antithrombotic drug using, or those who had received immunosuppressive treatment were excluded. Information regarding clinical pathological characteristics of patients was summarized in Table 1.

**Figure 3:**
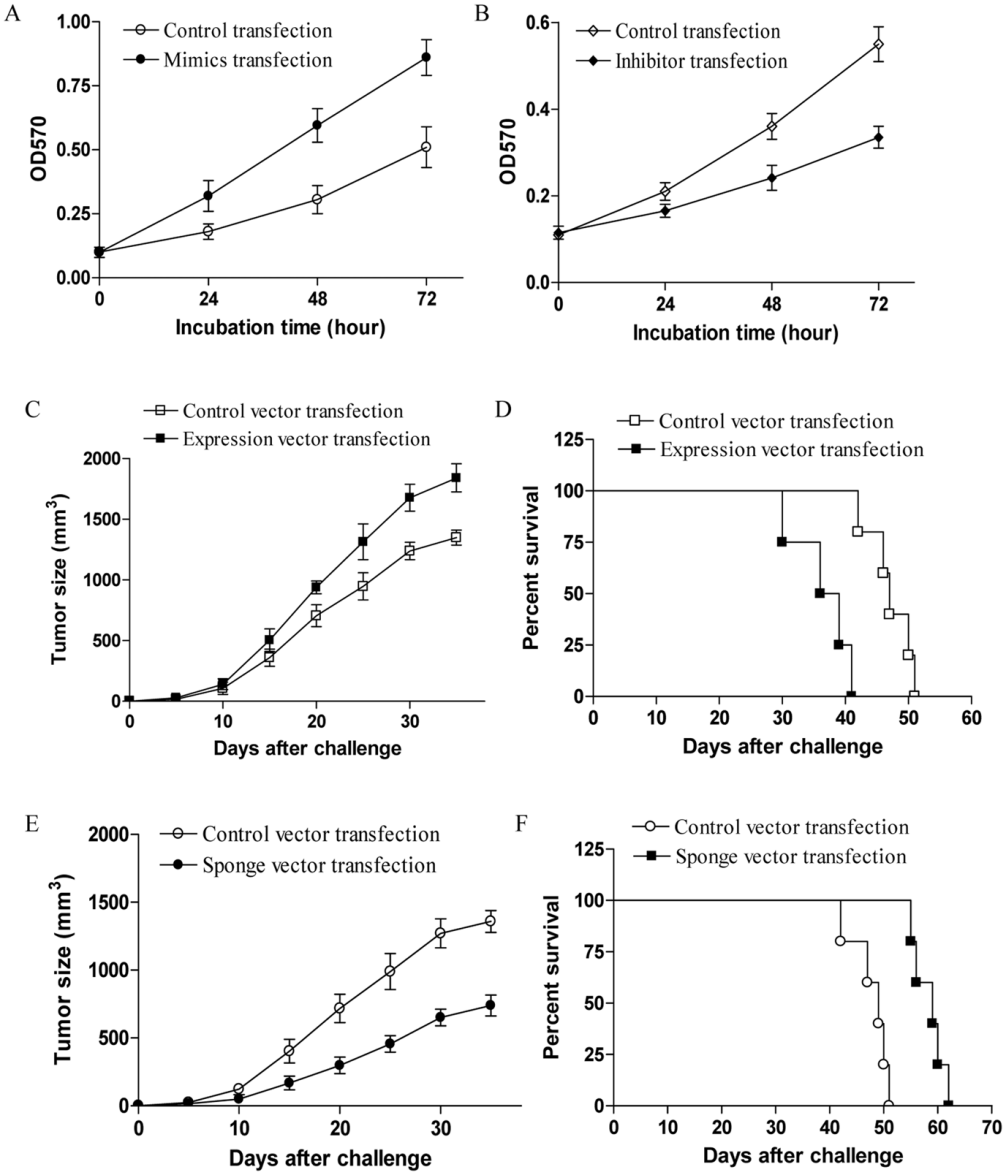
miR-574-59 promoted tumor progression of human lung cancer cells. (A) 95D cells were transfected with miR-574-5p mimics or the control respectively, and then assayed for their proliferation at the indicated time. (B) 95D cells were transfected with miR-574-5p inhibitor or the control respectively, and then assayed for their proliferation at the indicated time. Error bars indicated standard deviation of triplicate measurements form three independent experiments. (C–F) Groups of eight nude mice were challenged with 2×10^6^ of 95D cells which were stably transfected with miR-574-5p expression vector or miR-574-5p sponge vector respectively, and then assayed for their tumor progression in nude mice at the indicated time. The survival time of tumor bearing mice was also determined. Each bar represents the means (±SD) from eight nude mice in each group.

### Mice

Female BALB/c nude mice between 6 and 8 weeks of age were purchased from the Center of Experimental Animals of Tongji University. All mice were housed in a pathogen-free mouse colony at our institution.

**Figure 4:**
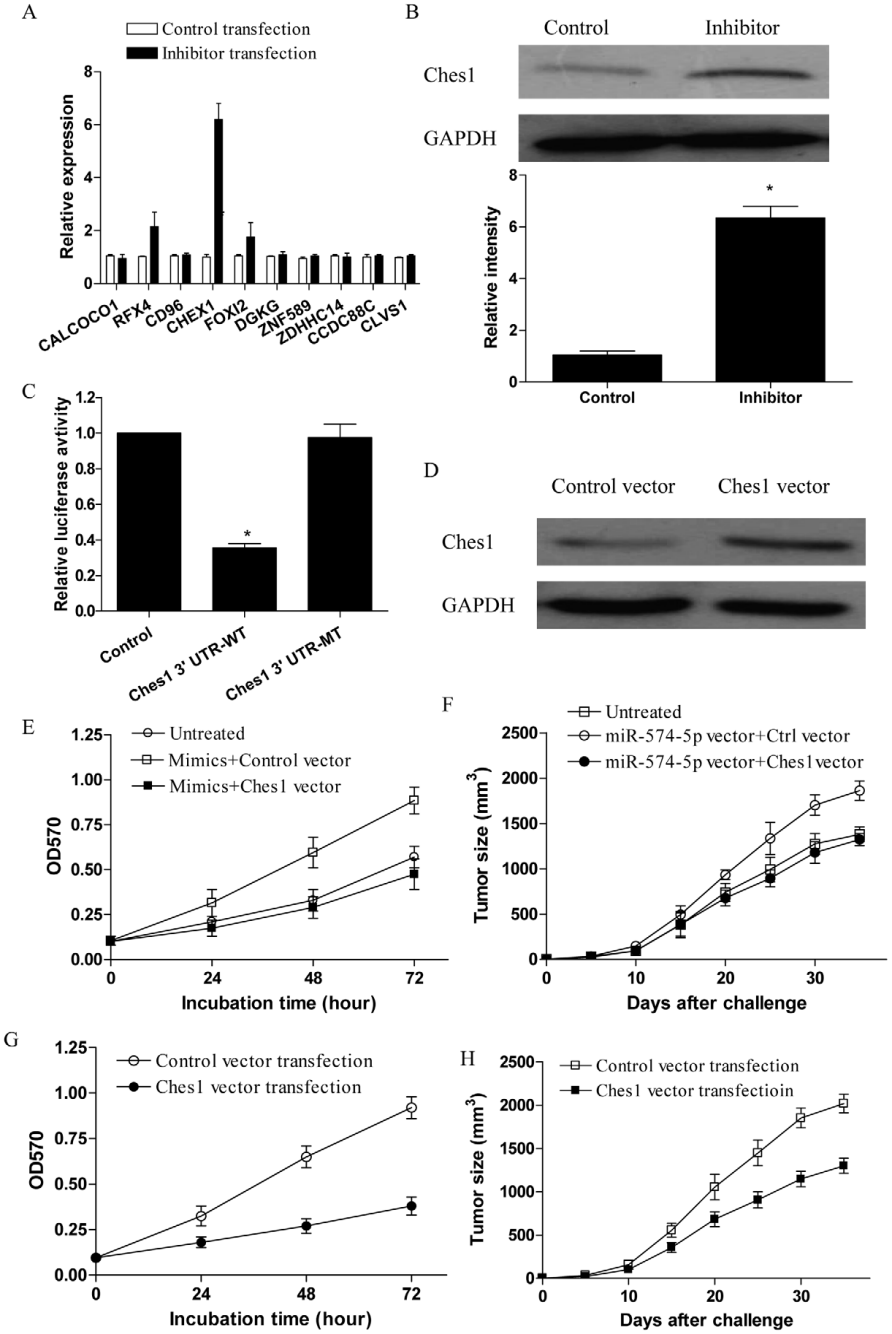
Ches1 was a direct target of miR-574-5p in regulating tumor progression of human lung cancer cells. (A) 95D cells were transfected with miR-574-5p inhibitor for 48 h and then assayed for their expression of the indicated genes using real time PCR. (B) 95D cells were transfected with miR-574-5p inhibitor for 48 h and then assayed for their expression of Ches1 using western blot. The relative intensity of Ches1 protein level from three independent experiments was shown. (C) 95D cells were co-transfected with miR-574-5p mimics and a luciferase reporter containing the wide type Ches1 3′ UTR or mutated Ches1 3′ UTR. (D) 95D cells were trasnfected with Ches1 expression vector for 48 h and then assayed for their expression of Ches1 protein level. (E) 95D cells were co-transfected with miR-574-5p mimics and Ches1 expression vector, and then assayed for their proliferation. (F) Groups of eight nude mice were challenged with 2×10^6^ of 95D cells which were stably transfected with miR-574-5p expression vector plus Ches1 expression vector or its control, and then assayed for their tumor progression at the indicated time. (G) 95D cells were transfected with Ches1 expression vector, and then stimulated with 10 µg/ml CpG ODNs for the indicated time. (H) Groups of eight nude mice were challenged with 2×10^6^ of 95D cells which were stably transfected with Ches1 expression vector or the control vector. Five days later, the tumor bearing mice were injected in situ with 100 µg of CpG ODNs at 7 day intervals. The tumor size was determined at the indicated time. Each bar represents the means (±SD) from eight nude mice in each group.

### Reagents and Cell Line

CpG ODN2216, control CpG ODN, chloroquine and the inhibitory peptide against MyD88 were purchased from InvivoGen. MiR-574-5p mimics and miR-574-5p inhibitor were purchased from Ribobio (Guangzhou, China). Annexin V-FITC apoptosis detection kit was purchased from eBioscience. Cell cycle phase determination kit was purchased from Cayman. The nucleofector kit was purchased from Amaxa. The human lung cancer cell line 95D cells was obtained from ATCC and maintained at 37°C under 5% CO_2_ in complete RPMI 1640 medium (GIBCO) containing 10% heat-inactivated fetal bovine serum supplemented with 2 mM glutamine, 100 IU/ml penicillin and 100 mg/ml streptomycin sulfate.

**Figure 5:**
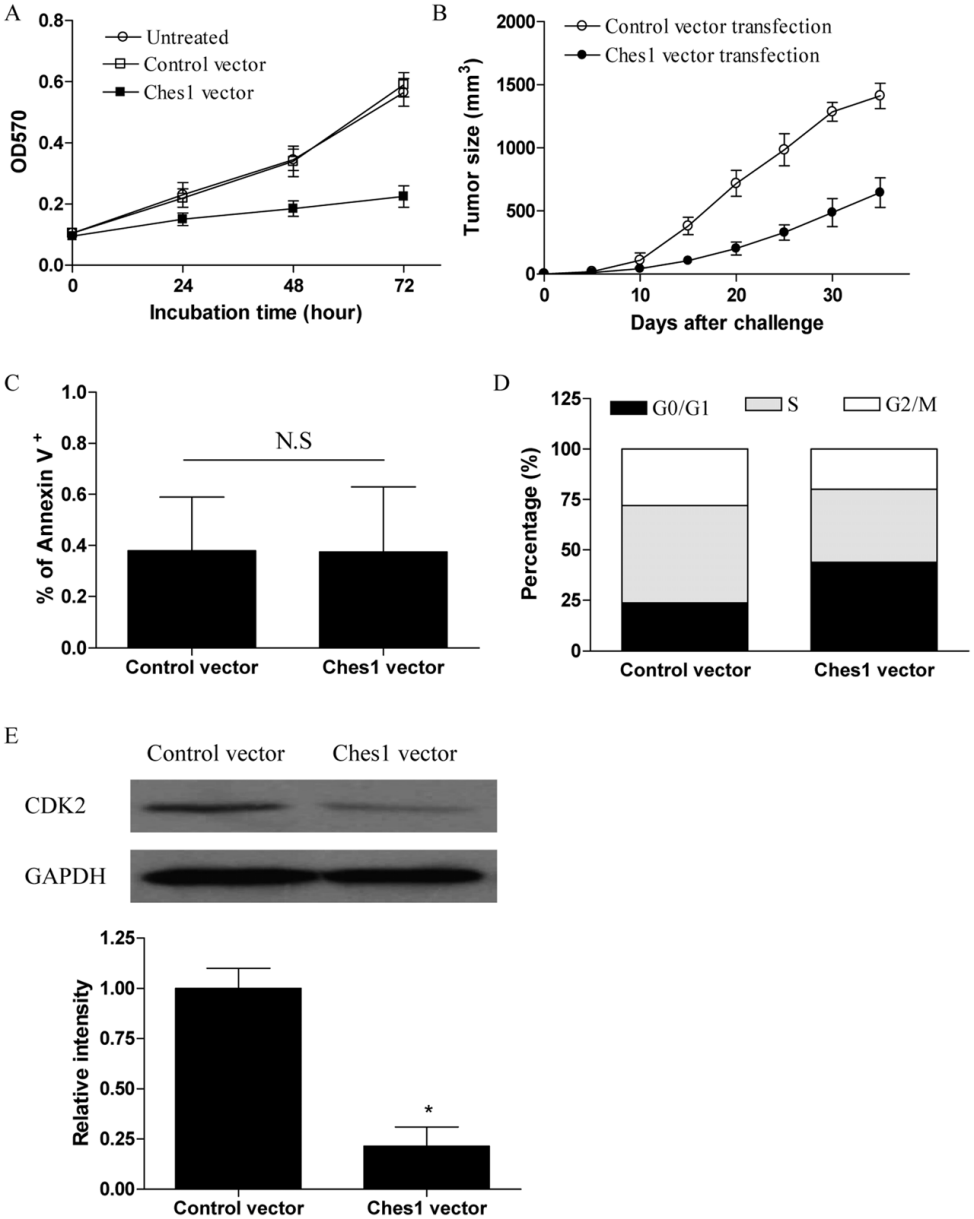
Over-expression of Ches1 inhibited cell cycle entry of human lung cancer cells. (A) 95D cells were transfected with Ches1 expression vector and assayed for their proliferation at the indicated time. (B) Groups of eight nude mice were challenged with 2×10^6^ of 95D cells which were stably transfected with Ches1 expression vector or the control vector. The tumor size was determined at the indicated time. Each bar represents the means (±SD) from eight nude mice in each group. (C and D) 95D cells which were stably transfected with Ches1 expression vector were first synchronized at the G0 phase by replacing the culture medium with serum-free medium for 24 hours, and detected for their apoptosis using Annexin V staining and cell cycle using the cell cycle phase determination kit by flow cytometry. (E) 95D cells were transfected with Ches1 expression vector and assayed for their CDK2 protein level 48 h later. Error bars indicated standard deviation of triplicate measurements from three independent experiments.*p<0.05.

### MTT Assay

95D cells were seeded at 3×10^3^ cells each well and incubated in the presence or absence of CpG ODN (10 µg/ml) in 96-well plates for 72 h. Assessment of cell proliferation was measured using commercially MTT cell proliferation kit (Cayman) according to the manufacture’s instructions.

**Figure 6:**
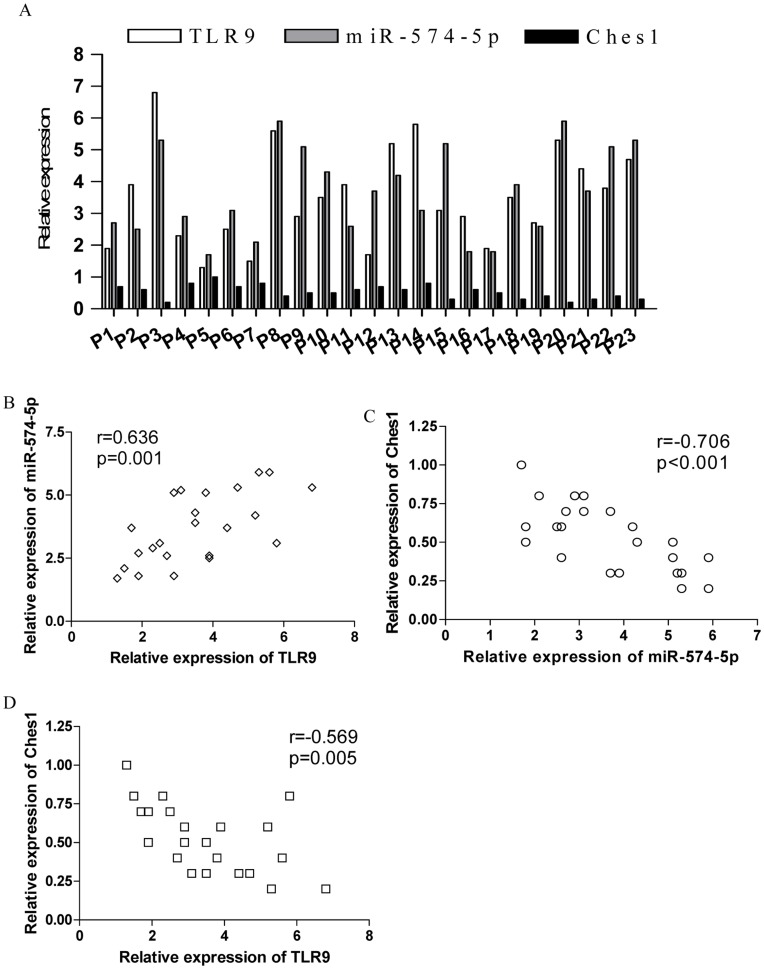
The expression of TLR9, miR-574-5p and Ches1 in clinical lung cancer. (A) Relative TLR9, miR-574-5p and Ches1 expression were determined by real time PCR in 23 NSCLC lung cancer samples. Each bar represents the relative ratio of these genes in cancer tissue versus adjacent lung tissue. Error bars indicated standard deviation of triplicate measurements form three independent experiments. (B–D) The correlation analyses between the expression of TLR9 and miR-574-5p, miR-574 and Ches1, as well as TLR9 and Ches1, were performed. Each dot represented the results from one patient.

### Real-time PCR

Quantitative Real-time RT-PCR was performed as previously described [20]. All the primers and probes were obtained from Applied Biosystems. Total RNA was extracted using TRIzol reagent. cDNA was synthesized with the PrimeScript RT reagent Kit (TaKaRa). Quantitative RT-PCR (qRT-PCR) analyses were carried out to detect mRNA expression using SYBR Premix Ex Taq (TaKaRa), and β-actin was used as an internal control. TaqMan micro-RNA assays (Applied Biosystems) were used to quantitative the expression levels of mature miR-574-5p, and U6 small nuclear RNA was used as an internal control.

### Western Blotting

Cells were lysed with M-PER Protein Extraction Reagent (Pierce) supplemented with a protease inhibitor cocktail. Cytoplasmic and nuclear extracts were prepared using NE-PER Nuclear and Cytoplasmic Extraction Reagents (Pierce). After centrifugation at 13,000 g under 4°C for 15 min, the supernatants were collected, and the protein concentration of the extracts was measured by BCA Protein Assay (Pierce) according to manufacturer’s instructions. Twenty micrograms of the protein were loaded onto 10% SDS-polyacrylamide gels and transferred for 90 min at 100 V onto polyvinylidene fluoride membranes using a wet transfer system. The membranes were washed in 5% skim milk in phosphate buffered saline plus 0.05% Tween 20 (PBST) for 2 h in order to block nonspecific protein binding sites on the membrane. Immunoblotting was performed using monoclonal antibodies to Ches1 and CDK2 (Sigma and Cell Signaling Technology) at a dilution of 1∶1000 in nonfat milk Tris buffer. The membrane was then washed in PBST, probed with a secondary anti-rabbit antibody conjugated to horseradish peroxidase (Amersham Life Sciences) at a dilution of 1∶5000, developed using an ECL Western Blotting KIT (Pierce), and exposed to X-ray film (Kodak).

### Plasmid Construction

Retrovirus-mediated overexpression or inhibition of miR-miR-574-5p was generated based on pMX vector (Invitrogen) as previously described [21]–[23]. Briefly, the miR-574-5p expression vector was constructed by ligating EcoRI/XhoI polymerase chain reaction (PCR) fragments with pMX-puro retroviral vector. Retroviral miR-574-5p “sponge” vector was constructed using ligation of two copies of miR-574-5p complementary oligos and pMX-puro vector. Sequence encoding mutant miR-574-5p was cloned into the same vector and used as the control vector. Virus was produced and target cells were infected according to the user's manual. Generation of Ches1 full-length plasmid was performed as previously described [24].

### Luciferase Reporter and Mutagenesis Assay

The luciferase reporter and mutagenesis assay were performed as previously described [20]. MiR-574-5p mimics or its control was co-transfected into 95D cells with a single report plasmid (pMIR-Report plasmid; Ambion) containing either the wild-type or mutated 3′ UTR of Ches1 which was generated using Quickchange Site-Directed Mutagenesis Kit (Stratagene). After 48 hours of transfection, firefly and Renilla luciferase activities were measured using the dual-luciferase reporter assay system (Promega).

### Evaluation of Tumor Growth in vivo

Evaluation of tumor growth was performed as described previously with minor modifications [3]. Briefly, BALB/c nu/nu mice (6–8 weeks old) were injected subcutaneously with 0.2 ml of a single-cell suspension containing 2×10^6^ tumor cells and kept in laminar flow cabinets under specific pathogen-free conditions. Tumors were measured every 5 days following tumor challenge using vernier calipers. Tumor volumes were obtained by multiplying the measured length by the measured width by the calculated mean of these measured values and were presented as the mean ± SEM.

### Statistical Analyses

Statistical analyses of the data were performed with the aid of analysis programs in SPSS12.0 software. Statistical evaluation was performed using two-way analysis of variance (ANOVA, p<0.05) using the program PRISM 4.0 (GraphPad Software Inc., San Diego, CA, USA).

## Results

### TLR9 Signaling Up-regulated the Expression of miR-574-5p in Human Lung Cancer Cells

We first confirmed the enhanced tumor progression of human lung cancer induced by TLR9 signaling, and found that CpG ODNs treatment of 95D cells significantly enhanced their proliferation in vitro and tumor progression in vivo (Figure 1A and B, p<0.05). To elucidate the potential role of miR-574-5p in the effects of TLR9 signaling on the progression of human lung cancer cells, we validated our previous microarray platform and assessed the expression of miR-574-5p in 95D cells with or without CpG ODNs treatment by real time PCR analysis. We revealed that the expression of miR-574-5p was indeed significantly elevated in 95D cells after CpG ODNs treatment in a dose and time dependent manner (Figure 1C and D, p<0.05). To further confirm that it was TLR9/MyD88 signaling that conferred the up-regulated expression of miR-574-5p, the TLR9 signaling inhibitor chloroquine and MyD88 inhibitory peptide were applied to block their signaling pathway. We found that both chloroquine and MyD88 inhibitory peptide could significantly abrogate the elevated expression of miR-574-5p induced by CpG ODNs in 95D cells in a dose dependent manner (Figure 1E and F, p<0.05). Our findings strongly demonstrated that TLR9 signaling effectively elevated the expression of miR-574-5p in human lung cancer cells.

### Down-regulation of MiR-574-5p Abrogated the Enhanced Tumor Progression Induced by TLR9 Signaling in Human Lung Cancer

To elucidate the potential role of miR-574-5p in the enhanced tumor progression of human lung cancer induced by TLR9 signaling, 95D cells were transfected with miR-574-5p inhibitor and then stimulated with CpG ODNs. As shown in Figure 2A, we found that transfection with miR-574-5p inhibitor effectively reduced their expression in 95D cells (p<0.05). Notably, we showed that transfection with miR-574-5p inhibitor significantly inhibited the proliferation of 95D cells induced by CpG ODNs (Figure 2B, p<0.5). To further confirm this phenomenon in vivo, miR-574-5p sponge was constructed to downregulate the expression of miR-574-5p. The level of functional knockdown of miR-574-5p was assayed by a reporter assay, in which the predicted miR-574-5p-binding site was introduced into the 3′ UTR of a luciferase reporter. We found that transfection of 95D cells with the miR-574-5p sponge caused significant increase in luciferase activity compared with the control sponge (Figure 2C, p<0.05), suggesting that effective inhibition of miR-574-5p activity was achieved. Thus, groups of nude mice were challenged with 95D cells which stably expressed miR-574-5p sponge, and then stimulated with CpG ODNs. Of note, we showed that down-regulation of miR-574-5p in 95D cells significantly inhibited their enhanced progression induced by TLR9 signaling in vivo (Figure 2D, p<0.05). Consistently, we found that down-regulation of miR-574-5p also significantly prolonged the survival time of tumor bearing nude mice (Figure 2E, *p*<0.05). Combining these data suggested that miR-574-5p conferred the effect of TLR9 signaling on tumor progression of human lung cancer cells.

### MiR-574-5p Promoted the Tumor Progression of Human Lung Cancer Cells

To characterize the mechanism underlies the pivotal role of miR-574-5p in enhanced tumor progression induced by TLR9 signaling, we further evaluated the direct effect of miR-574-5p on the growth of 95D cells in vitro and in vivo. As shown in Figure 3A, we found that over-expression of miR-574-5p in 95D cells resulted in their elevated proliferation in vitro (p<0.05). Consistently, reduced expression of miR-574-5p in 95D cells impaired their proliferation in vitro (Figure 3B, p<0.05). To further confirm these results in vivo, nude mice were challenged with 95D cells which were stably transfected with miR-574-5p expression vector or miR-574-5p sponge vector respectively. We found that over-expressed miR-574-5p significantly enhanced the tumor progression of 95D cells in vivo, accompanied by a reduced survival time of tumor bearing mice (Figure 3C and D, p<0.05). Consistently, down-regulation of miR-574-5p in 95D cells abrogated their growth in vivo and prolonged the survival time of tumor bearing mice (Figure 3E and F, p<0.05). These results suggested that miR-574-5p was an important factor associated with enhanced tumor progression of human lung cancer.

### Ches1 was a Direct Target for miR-574-5p to Promote Tumor Progression of Human Lung Cancer

To further understand the effect of miR-574-5p on regulating the tumor progression of human lung cancer cells, we predicted the targets of miR-574-5p by prediction programs including TargetScan and Miranda, and selected 10 possible target including CALCOCO1, RFX4, CD96, CHEX1, FOXI2, DGKG, ZNF589, ZDHHC14, CCDC88C, CLVS1 for real time PCR analysis. We found that the expression of Ches1 exhibited a dramatically elevation in 95D cells transfected with miR-574-5p inhibitor (Figure 4A, p<0.05). To confirm this result, we performed western blot to detect the expression of Ches1 in 95D cells transfected with miR-574-5p inhibitor. We found that the protein level of Ches1 was significantly increased by transfection with miR-574-5p inhibitor in 95D cells (Figure 4B, p<0.05). We then performed a luciferase reporter assay to validate the potential regulatory relationship. A significantly negative effect on luciferase activity was observed on the 3′ UTR of Ches1 in the presence of miR-574-5p, such repression disappeared when the predicted target site in the 3′ UTR of Ches1 was mutated (Figure 4C, p<0.05). To further detect the potential role of Ches1 in TLR9 signaling enhanced growth of 95D cells, 95D cells were transfected with Ches1 expression vector and then stimulated with CpG ODNs. As shown in Figure 4D, we found that transfection with Ches1 expression vector significantly increased their expression in 95D cells (p<0.05). Notably, we found that transfection of miR-574-5p mimics failed to enhance the proliferation of 95D cells which were co-transfected with the Ches1 expression vector (Figure 4E, p<0.05). We further showed that transfection with Ches1 expression vector effectively abrogated the tumor promoting effect of miR-574-5p in vivo (Figure 4F, p<0.05). Furthermore, we revealed that up-regulation of Ches1 effectively inhibited the TLR9 signaling enhanced proliferation of 95D cells (Figure 4G, p<0.05). Consistently, we found that over-expression of Ches1 in 95D cells effectively impaired their enhanced tumor progression induced by TLR9 signaling in nude mice (Figure 4H, p<0.05). These results indicated that Ches1 was a bona fide target of miR-574-5p in regulating TLR9 signaling enhanced tumor progression of human lung cancer.

### Over-expression of Ches1 Inhibited Cell Cycle Entry of Human Lung Cancer Cells

To elucidate the potential role of Ches1 in the tumor progression of human lung cancer cells, 95D cells stably transfected with Ches1 expression vector were detected for their proliferation in vitro and progression in nude mice in vivo. We showed that over-expression of Ches1 significantly inhibited the proliferation of 95D cells (Figure 5A, p<0.05). Consistently, we found that the tumor progression of 95D cells transfected with Ches1 expression vector was dramatically ameliorated than the control group (Figure 5B, p<0.05). These findings suggested that Ches1 was an inhibitory player in the growth of human lung cancer cells. To further characterize the underlying mechanism, we analyzed the possible effect of Ches1 on the apoptosis and cell cycle of human lung cancer cells. 95D cells stably transfected with Ches1 expression vector or the control vector were first synchronized at the G0 phase by replacing the culture medium with serum-free medium, and then continuously cultured and harvested after 24 hours. We found that the apoptosis rate of 95D cells transfected with Ches1 expression vector was low and generally comparable with the control group (Figure 5C). In contrast, the percentage of 95D cells transfected with Ches1 expresson vector at Go/G1 stage was significantly higher than those in the control group (Figure 5D, p<0.05). Besides, we also revealed the reduction of CDK2 expression in 95D cells after transfection with Ches1 expression vector (Figure 5E, p<0.05), which could partly explain our results and was consistent with our previous study demonstrating that up-regulation of CDK2 was critical for TLR9 signaling to stimulate the proliferation and cell cycle entry of human lung cancer cells [4].

### The Expression of miR-574-5p was Positively Correlated with TLR9 and Reversely Correlated with Ches1 in Clinical Lung Cancer Patients

To further investigate the potential role of miR-574-5p in the effect of TLR9 signaling on human lung cancer patients, we detected the relationship between the expression of miR-574-5p and TLR9 in clinical NSCLC patients. As shown in Figure 6A, the expression levels of TLR9 and miR-574-5p were higher in lung cancer tissue compared with adjacent tissue of tumor from clinical lung cancer samples (p<0.05). In contrast, the expression of Ches1 was significantly lower in tumor tissues compared with adjacent tissues (Figure 6A, p<0.05). Importantly, we found that the expression level of miR-574-5p was positively correlated to the expression of TLR9 in clinical tumor tissues (Figure 6B, p<0.05). Furthermore, we revealed that the expression of miR-574-5p was reversely correlated with the expression level of Ches1 in the tumor tissues (Figure 6C, p<0.05). Moreover, the expression of TLR9 was also reversely correlated with the expression level of Ches1 in the tumor tissues (Figure 6D, p<0.05). These findings were in line with our above data which demonstrated that Ches1 was a predominate target for miR-574-5p to confer the enhanced tumor progression induced by TLR9 signaling in human lung cancer.

## Discussion

In recent years, accumulating data suggested that TLRs were functional expressed in tumor cells and involved in tumor progression [4]–[8]. We previously demonstrated that TLR9 signaling could enhance the tumor progression of human lung cancer cells in vitro and in vivo [3], [4], [9]–[11]. Here we extended our previous study by demonstrating that up-regulation of miR-574-5p conferred the enhanced tumor progression induced by TLR9 signaling in human lung cancer cells. Our findings suggested that miR-574-5p was an important player in TLR9 signaling and tumor biology.

In present study, we found that TLR9 signaling significantly elevated the expression of miR-574-5p in 95D cells, which was consistent with our previous study [19]. To address the potential role of miR-574-5p in TLR9 signaling enhanced progression of human lung cancer cells, we evaluated the effect of down-regulation of miR-574-5p on TLR9 signaling enhanced progression of 95D cells and found that down-regulation of miR-574-5p could obviously reduce the progression of 95D cells in vitro and in vivo. Our data suggested that intrinsic miR-574-5p could contribute to the progression of human lung cancer cells. Indeed, our successive study showed that miR-574-5p could promote the progression of human lung cancer cells, indicating that sustained expression of miR-574-5p was pivotal for the progression of human lung cancer cells. Consistently, we further showed that the expression of TLR9 and miR-574-5p was higher in clinical tumor tissues and exerted a positively correlation. Our data were in line with previous study which demonstrated that the expression of miR-574-5p was significantly increased in NSCLC samples with respect to the controls [25]. Combing all these findings suggested that miR-574-5p might be an optimistic candidate for diagnosis, prediction and treatment of human lung cancer. However, the precise mechanism for how TLR9 signaling activated miR-574-5p expression still remains unclear.

Ches1 is a member of the forkhead family of transcription factors [26]. This molecule was discovered based on its ability to inhibit cell division by causing cell cycle arrest after DNA damage in yeast cells [26], [27]. Ches1 has also been reported as a component of a transcriptional repressor complex, which included Sin3a and HDAC to interact with MEN1 protein in response to DNA damage [24], [26]. Recently, accumulating expression profile data suggested that Ches1 played a role in tumorigenicity and responses to cancer treatments [24], [28]–[30]. In this study, we identified Ches1 as the direct target of miR-574-5p in regulating the progression of human lung cancer cells. We found that over-expression of Ches1 effectively impaired the tumor progression of human lung cancer cells. Furthermore, we revealed that Ches1 was an important player in regulating the cell cycle entry of human lung cancer cells. Our findings were in line with recent data which showed that over-expression of Ches1 suppressed cell growth and arrested cell cycle of oral cancer cells [24]. However, the precise mechanism underlies the effect of Ches1 on the cell cycle entry of human lung cancer cells undoubtedly needed successive studies.

Our previous study revealed that TLR9 signaling could contribute to the metastasis of human lung cancer [9]–[11]. Here we found that miR-574-5p could enhance the tumor progression of 95D cells in vivo, indicating that miR-574-5p might be also involved in the metastasis of human lung cancer cells, which remains to be elucidated. Besides, the important role of miR-574-5p in TLR9 signaling did not exclude other miRNAs that might be involved in TLR9 signaling in human lung cancer. In fact, we found that miR-7 was also an important player in TLR9 signaling in human lung cancer (unpublished data). However, the possible interaction or crosstalk between miRNAs and TLRs still remains unclear. In addition, it should be pointed out that although we confirmed our findings in A549 cells and found similar results in vitro and in vivo (data not shown), additional research on more human cancer cell lines, human lung cancer tissues and other tumor tissues still deserved successive studies.

In conclusion, here we extended previous study by demonstrating that miR-574-5p was pivotal for TLR9 signaling enhanced tumor progression via down-regulating Ches1 in human lung cancer. Our findings not only facilitated the further understanding the crosstalk between miRNAs and TLRs in tumor biology, but they also provided novel potential candidates for treatment of cancer.
